# Spatiotemporal Dynamics of Bacterial Community Assembly and Co-Occurrence Patterns in Biological Soil Crusts of Desert Ecosystems

**DOI:** 10.3390/microorganisms13020446

**Published:** 2025-02-18

**Authors:** Runze Bao, Kai Tang, Yanfu Ji, Shengnan Zhang, Chunying Wang, Yungang Liang, Xiujuan Zhao, Jianyu Meng

**Affiliations:** 1 Laboratory for Environmental Microbiology and Biotechnology in Arid and Cold Regions, College of Life Sciences, Inner Mongolia Agricultural University, Hohhot 010018, China; runzebao@126.com (R.B.); htangkai@126.com (K.T.); c_33565014@163.com (Y.J.); lygaem@163.com (Y.L.); 2 Inner Mongolia Duolun Hunshandake Sandland Ecosystem National Observation and Research Station, Inner Mongolia Academy of Forestry Sciencese, Hohhot 010010, China; nmzhangshengnan@163.com (S.Z.); wangcy_1985@163.com (C.W.)

**Keywords:** biological soil crusts, bacterial communities, β-NTI, community assembly, environmental filtering, network analysis

## Abstract

Biological soil crusts (BSCs) play a fundamental role in desert ecosystems by stabilizing soil, cycling nutrients, and retaining moisture. However, the assembly processes governing bacterial communities within BSCs remain largely unknown. This study aimed to reveal the spatiotemporal variations in the bacterial community diversity, co-occurrence patterns, and ecological assembly processes of BSCs and their underlying soils across different desert and seasonal conditions. We systematically analyzed the spatial differences in the bacterial diversity, co-occurrence networks, and community assembly processes of BSCs and their underlying soils using samples collected at various soil depths from different BSC types in different deserts. We discovered that BSC type and soil depth were the primary factors driving bacterial community assembly, while seasonal effects were weaker and more indirect, and mainly regulated community dynamics through changes in resource availability and environmental conditions. The underlying soils of moss- and lichen-BSCs exhibited higher bacterial diversity and richness than those of algae BSCs. In contrast, cyano-BSCs exhibited a lower diversity, but Cyanobacteria demonstrated the highest photosynthetic function. Among the different deserts, the community assembly of samples from the eastern Inner Mongolia deserts was largely influenced by environmental selection, whereas stochastic processes were more prominent in the central and western desert regions. A β-nearest taxon index (βNTI) analysis indicated that stochastic processes were dominant in surface BSC samples, while environmental selection played a stronger role in deeper layers. A co-occurrence network analysis revealed that surface BSC samples had a high degree of network connectivity, with those from moss- and lichen-BSCs being particularly high, and they also exhibited high modularity and local clustering that promoted the functional stability of the microbial communities. This study revealed the integrated effects of soil depth, BSC type, desert type, and resource availability on microbial community assembly in desert ecosystems. These findings provide a theoretical basis for the microbial management of BSCs and scientific insights to support restoration strategies in desert ecosystems.

## 1. Introduction

Biological soil crusts (BSCs) are complex consortia composed of Cyanobacteria, mosses, lichens, and other microorganisms that are crucial to arid and semi-arid ecosystems [1]. BSCs contribute significantly to soil stability, nutrient cycling, and moisture retention, and thereby support desert ecosystem functions [2,3,4]. The microbial communities within BSCs are highly dynamic and influenced by various environmental factors, such as soil nutrient levels, moisture availability, and seasonal changes [5]. Understanding the composition and assembly processes of these microbial communities is essential to elucidating their ecological functions and adaptation strategies in extreme semi-arid environments.

Microbial community assembly processes are generally driven by two major mechanisms: deterministic processes (e.g., environmental filtering and species interactions) and stochastic processes (e.g., drift, dispersal, and random colonization) [6,7]. Deterministic processes tend to produce predictable patterns along environmental gradients, whereas stochastic processes introduce randomness into the community composition [8]. The balance between these two processes determines the structure and function of the microbial communities [9]. In arid and semi-arid environments such as deserts, resource availability is highly variable, temperature fluctuations are extreme, and spatial heterogeneity is pronounced [10,11,12]; therefore, understanding the relative contributions of deterministic and stochastic processes is a significant challenge.

Research on BSC microbial communities provides valuable information on how microorganisms adapt to harsh environments and how environmental factors influence community assembly. Deserts are characterized by nutrient scarcity, limited water resources, and extreme temperatures, all of which impose strong selective pressures on microbial communities [13,14,15]. Moreover, in desert environments, microbial BSCs are less influenced by plants and animals than in many other environments, resulting in relatively independent and simple microbial communities [16,17,18]. Therefore, BSC samples from desert environments can serve as ideal “model samples” for studying BSC microbiota. In this context, understanding the assembly processes of BSC microbial communities can provide insights into the resilience and functional stability of desert ecosystems. Additionally, elucidating the microbial assembly processes of BSCs can inform ecological restoration and desertification control, particularly by identifying key environmental drivers that enhance microbial diversity and ecosystem processes.

We investigated the assembly processes of bacterial communities in BSCs and their subsoils in three desert regions in northern China: Hunshandake Desert, Kubqi Desert, and Tengger Desert. These regions represent a range of environmental conditions ranging from relatively humid to extremely arid [19], providing a unique opportunity to explore spatial and temporal variations in bacterial community assembly. In this study, our objectives were to answer the following questions: (1) How do environmental factors influence the bacterial community structure in these desert areas? (2) How do bacterial community characteristics affect diversity, co-occurrence patterns, and assembly metrics? Based on these questions, we hypothesize that the assembly of bacterial communities in biological soil crusts (BSCs) is primarily influenced by a combination of environmental factors, including BSC type, soil depth, and geographical location, with seasonal variations playing a secondary role. We further hypothesize that environmental selection processes will dominate community assembly in the subsoil layers, while stochastic processes will have a stronger influence in surface layers where resources and environmental conditions fluctuate more frequently. By addressing these questions, we aim to deepen our understanding of the ecological processes governing bacterial community dynamics in desert environments, which has important implications for desertification management and degraded ecosystem restoration.

## 2. Experimental Procedures

### 2.1. Sample Collection, Soil Physicochemical Analysis, DNA Extraction, and 16S rRNA Amplicon Sequencing

The study sites were located in the Hunshandake Desert, Kubqi Desert, and Tengger Desert, representing the eastern, central, and western regions, respectively, of the eight major sandy areas of Inner Mongolia (elevation, longitude, latitude, mean annual temperature, and mean annual precipitation are provided in Table S1). In May 2015 (wet season) and September 2016 (dry season), samples of Cyanobacterial crusts (cyano-BSCs), lichen crusts (lichen-crusts), and moss crusts (moss-crusts), along with samples of the corresponding subsoil (2 cm below the BSCs), were collected from five sites. These two time points were selected to capture the most representative seasonal variations in the desert ecosystem. A total of 50 samples were collected, comprising 14 samples from cyano-crusts, 16 from lichen-crusts, and 20 from moss-crusts, including their respective subsoil layers.

The sample collection and subsequent DNA extraction, amplicon sequencing, and soil physicochemical analysis methods in this study were all based on a study by Tang Kai et al. (2021) [20].

### 2.2. Bacterial Community Assembly Analysis

The β-nearest taxon index (βNTI) was used to quantify community assembly. To determine whether the bacterial communities in the BSCs and their subsoils were shaped by deterministic or stochastic processes, the βNTI was calculated using the Picante package in R (1.8.2) [6]. A null model was generated by 999 randomizations based on observed data (operational taxonomic unit [OTU] table and phylogenetic tree), and the βNTI was calculated as the standard deviation of the difference between the observed β-mean-nearest-taxon-distance (β-MNTD) and the mean β-MNTD of the null model. A βNTI > 2 indicated that the observed β-MNTD was significantly different from that obtained by the random simulation, suggesting that deterministic processes predominantly shaped the community changes. Specifically, a βNTI < −2 indicated homogeneous selection, and a βNTI > 2 indicated heterogeneous selection [6]. A βNTI < 2 indicated that community changes were mainly due to stochastic processes. For a βNTI < 2, Raup–Crick Bray–Curtis (RCbray) values > 0.95 or < −0.95 indicate that homogenizing dispersal and dispersal limitation, respectively, influence community variation, and an RCbray value < 0.95 indicates that community changes are not dominated by a single process.

### 2.3. Co-Occurrence Network Analysis

The construction and analysis of the bacterial molecular networks were based on the Molecular Ecological Network Analysis Pipeline [21]. Spearman’s correlation analysis was used to assess the OTU abundance of bacteria to obtain correlation coefficients (r) and significance values (*p*) [22]. A threshold value was generated based on the random matrix theory [23], and an adjacency matrix was constructed from the correlation coefficient matrix to determine the nodes and edges of the co-occurrence network. Co-occurrence networks were visualized using Gephi v 0.9.2.

### 2.4. Statistical Analysis

One-way analysis of variance (ANOVA) was used to evaluate differences in the bacterial α-diversity among the samples, followed by Tukey’s post hoc test [24]. Principal coordinate analysis (PCoA) was used to assess differences in bacterial community structures among samples [25]. Statistical analyses and visualization were performed using R v.4.1.3 (R Development Core Team, Vienna, Austria).

Structural equation modeling (SEM) using the “piecewiseSEM” R package (2.3.0.1) was used to construct and fit models to investigate the direct and indirect effects of different environmental factors (such as soil depth and seasonality) on bacterial community assembly in the BSCs [26]. Initially, an a priori model was developed based on known relationships among variables related to soil properties, microbial diversity, and the βNTI from the existing literature and using expert knowledge. To improve the stability and goodness of fit of the model, potential variables and pathways were initially selected to exclude predictors that contributed poorly to the model fit. Model fit was assessed using the Akaike information criterion [27].

## 3. Results

### 3.1. Bacterial Diversity in BSCs Across Soil Layers and Desert Regions

**Figure 1:**
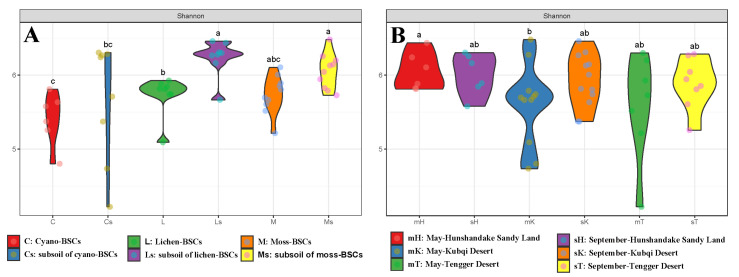
Comparison of Shannon diversity indices across biological soil crust (BSC) types, subsoils, and sampling regions. (**A**) Shannon diversity indices of different BSC types, including cyano-BSCs (C), lichen-BSCs (L), moss-BSCs (M), and their respective subsoils (Cs, Ls, Ms). Different letters above the violin plots indicate significant differences among groups (*p* < 0.05). (**B**) Shannon diversity indices across sampling regions and seasons: May and September samples from Hunshandake Sandy Land (mH, sH), Kubuqi Desert (mK, sK), and Tengger Desert (mT, sT). Different letters indicate significant differences (*p* < 0.05).

In the surface BSC samples, the different types of BSCs exhibited significant differences in microbial diversity. The Shannon diversity indices of the moss BSC and lichen BSC subsoil samples were significantly higher than those of the other BSC and subsoil samples (Figure 1A). The surface BSC samples of the moss- and lichen-BSCs showed similar diversity, with no significant differences between them. Both the surface and subsoil samples of the cyano-BSCs exhibited relatively low diversity, although the subsoil diversity was significantly higher than the surface diversity. Among the different sampling sites, the microbial diversity in the BSCs from the Hunshandake and Kubuqi Desert showed significant spatial variation (Figure 1B). Seasonal factors had no significant effect on bacterial diversity in the BSCs or their subsoil layers. Species richness, estimated by Chao1 and ACE indices, was higher in the subsoil layers than in the corresponding surface BSC samples for all BSC types (Figure S1), except for cyano-BSCs, which exhibited a lower richness. Moss-BSCs consistently showed the highest diversity and richness among the BSCs. Additionally, samples from the Hunshandake Desert exhibited lower diversity indices than those from the Kubuqi and Tengger Deserts (Figure S2).

A PCoA revealed distinct microbial community compositions between the surface and subsoil layers. The surface BSC samples were primarily located along the positive x-axis and subsoil samples along the negative x-axis (Figure S3). The PCoA results based on region and sampling time (Figure S4) showed clear separation of samples from the Hunshandake, Kubuqi, and Tengger Deserts, indicating strong geographic influences on microbial communities. No significant differences were observed between the samples collected in May and September, particularly in the Hunshandake and Kubuqi Desert, suggesting limited seasonal effects on microbial community structure.

### 3.2. Variations in the Composition of BSC Bacteria Among Different Soil Layers, Regions, and Seasons

**Figure 2:**
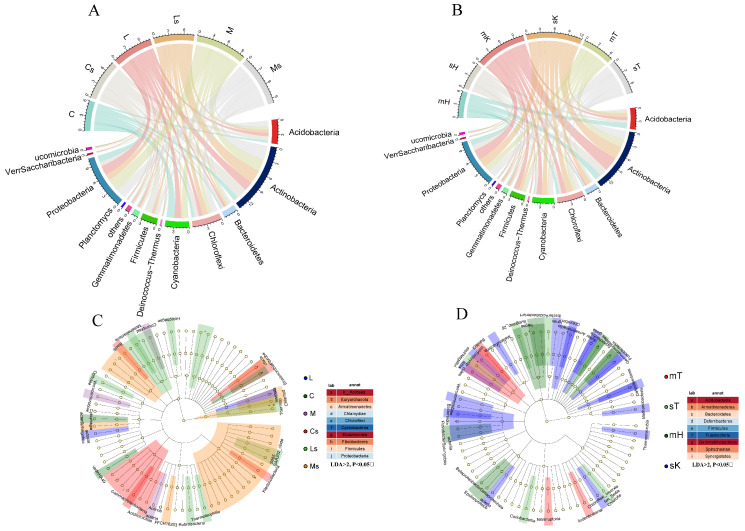
Microbial community composition and taxonomic distribution across biological soil crusts (BSCs), soil layers, and sampling regions. (**A**) Chord diagram showing the distribution of bacterial phyla across BSC types. (**B**) Chord diagram showing the distribution of bacterial phyla across sampling regions. (**C**) LEfSe analysis highlighting significantly enriched bacterial taxa across BSC types at the class level. (**D**) LEfSe analysis highlighting significantly enriched bacterial taxa across sampling regions at the class-level taxa enriched in specific conditions are indicated with statistical significance (LDA > 2, *p* < 0.05). Labels: cyano-BSCs (C), lichen-BSCs (L), moss-BSCs (M), and their respective subsoils (Cs, Ls, Ms); May and September samples from Hunshandake Sandy Land (mH, sH), Kubuqi Desert (mK, sK), and Tengger Desert (mT, sT).

At the phylum level, the microbial community composition varied significantly between the surface and subsoil samples (Figure 2A and Figure S5). In the surface BSC samples, Cyanobacteria were dominant, particularly in cyano-BSCs from the Hunshandake Desert, where their abundance reached 68%. Moss-BSCs had a lower Cyanobacterial abundance but higher proportions of Actinobacteria (reached 30.42%) and Proteobacteria (reached 14.26%). In the moss BSC subsoil from the Kubqi and Tengger Deserts, Firmicutes were more abundant, and there was a significant increase in Actinobacteria and decrease in Cyanobacteria. The different BSCs also exhibited distinct microbial compositions. Moss-BSCs had higher abundances of Proteobacteria and Actinobacteria, while cyano-BSCs were dominated by Cyanobacteria. Lichen-BSCs showed a more balanced proportion of Cyanobacteria, Actinobacteria, and Proteobacteria. Geographic location influenced microbial composition, with Cyanobacteria more abundant in the Kubqi Desert, and Proteobacteria and Firmicutes more abundant in the Tengger Desert (Figure 2B and Figure S6). Seasonal variations were associated with a higher abundance of Firmicutes and Cyanobacteria in May, and of Proteobacteria in September. An LEfSe analysis at the class level highlighted significant differences in the microbial composition among the types of BSCs (Figure 2C), with Clostridia (Firmicutes) dominating moss-BSCs, which were also enriched in *Alphaproteobacteria* and *Gammaproteobacteria*. Actinobacteria was prevalent in lichen-BSCs. The surface BSCs showed significant enrichment of Cyanobacteria, and the abundance of Clostridia increased in the subsoil. Seasonal variations also affected the microbial composition; Cyanobacteria was were abundant in May and Clostridia and Actinobacteria increased in September (Figure 2D). The geographic location also influenced microbial composition. Actinobacteria was more abundant at sites with a higher organic carbon content, and Cyanobacteria was were enriched at sites with increased light and surface moisture. These results highlighted the combined influence of BSC type, soil depth, geographic location, and season on microbial community composition.

### 3.3. Co-Occurrence Patterns of Bacterial Communities in BSCs

**Figure 3:**
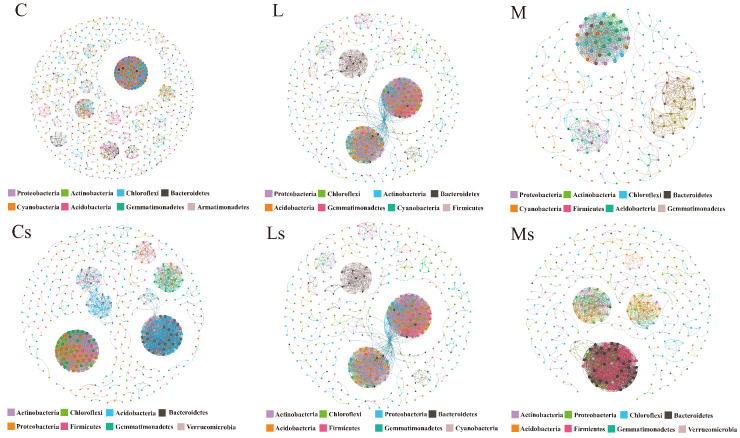
Co-occurrence network analysis of bacterial communities across different biological soil crust (BSC) types and their subsoils. The networks represent (C) cyano-BSCs, (Cs) subsoil of cyano-BSCs, (L) lichen-BSCs, (Ls) subsoil of lichen-BSCs, (M) moss-BSCs, and (Ms) subsoil of moss-BSCs. The nodes represent bacterial taxa at the phylum level, and edges represent significant correlations between taxa (Spearman’s correlation, *p* < 0.001, |r| > 0.80). The node colors indicate different bacterial phyla, while edge thickness corresponds to correlation strength. The networks illustrate differences in complexity and modularity between surface and subsoil samples for each BSC type.

A network analysis revealed significant differences between the microbial co-occurrence patterns of the surface and subsoil layers (Table S2). Surface BSC samples, particularly cyano-BSCs, exhibited higher network complexity with more nodes and edges, indicating strong cooperative interactions, than subsurface samples. In contrast, the subsoil samples had higher average path lengths and clustering coefficients than the surface samples, suggesting more localized microbial interactions. Among the different BSC types, cyano-BSCs showed greater symbiotic relationships, while moss-and lichen-BSCs exhibited higher clustering effects, which may contribute to stabilizing microbial functions in arid conditions. Seasonal and spatial differences were also evident, with samples collected in May showing high network complexity, while those from September exhibited more dispersed networks than the samples from May. The cyano-BSC surface networks were highly centralized and dominated by Cyanobacteria, while the cyano-BSC subsoil networks had more complex modularity. The lichen-BSC surface networks were dispersed as independent bacterial clusters, while the subsoil networks were more modular, particularly the Proteobacteria and Actinobacteria subnetworks. Moss-BSCs showed complex networks in the surface BSC samples, with Actinobacteria and Proteobacteria forming key clusters, while the subsoil samples exhibited greater modularity, particularly of Actinobacteria and Acidobacteria (Figure 3). Network structures also varied by desert type and season, with distinct co-occurrence patterns observed in samples from the Hunshandake, Kubuqi, and Tengger Deserts (Figure S7). These results highlighted the dynamic and complex microbial interactions in BSCs under different environmental conditions.

### 3.4. Ecological Assembly Processes of Bacterial Communities in BSCs

**Figure 4:**
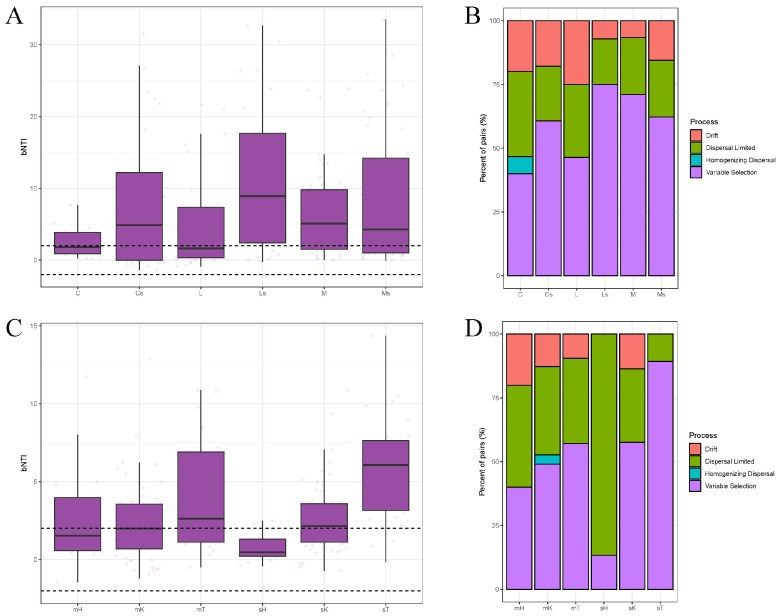
Patterns of microbial community assembly processes in biological soil crusts (BSCs) across different BSC types, regions, and seasons. (**A**) Boxplot of β-nearest taxon index (βNTI) values across different BSC types and their subsoils, where values > 2 or <−2 indicate dominance of deterministic processes, while values between −2 and 2 suggest stochastic processes, the dashed lines represent the positions of βNTI values of 2 and −2. (**B**) Relative contribution of community assembly processes (variable selection, homogeneous selection, dispersal limitation, homogeneous dispersal, and drift) across BSC types. (**C**) Boxplot of βNTI values across different regions and seasons. (**D**) Relative contribution of community assembly processes in different regions and seasons. Labels: cyano-BSCs (C), lichen-BSCs (L), moss-BSCs (M), and their respective subsoils (Cs, Ls, Ms); May and September samples from Hunshandake Sandy Land (mH, sH), Kubuqi Desert (mK, sK), and Tengger Desert (mT, sT).

The βNTI values (Figure 4A) indicated significant differences in the microbial community assembly between the different types of BSCs and underlying soil layers. The surface BSC samples predominantly had βNTI values within the range of −2 to 2, suggesting a balance between stochastic processes and environmental selection. In contrast, the subsoil samples had a wider range of βNTI values, indicating stronger environmental selection. The RCbray results (Figure 4B) further confirmed these patterns, suggesting that stochastic processes dominated in cyano-BSCs, and variable selection was the primary driver in lichen- and moss-BSCs. Geographic location also influenced community assembly. The samples from the Hunshandake Desert had higher βNTI values, reflecting stronger environmental selection pressures, while samples from the Kubqi and Tengger Deserts were more influenced by stochastic processes, particularly in September (Figure 4C). Seasonal differences were also observed, with variable selection dominating in May during the dry season and stochastic processes increasing in September as water availability became high (Figure 4D). Overall, microbial community assembly in the BSCs was influenced by a combination of stochastic processes and environmental selection, with the relative contributions of each factor varying under different environmental conditions.

### 3.5. Factors Influencing the Assembly of Bacterial Communities in BSCs

**Figure 5:**
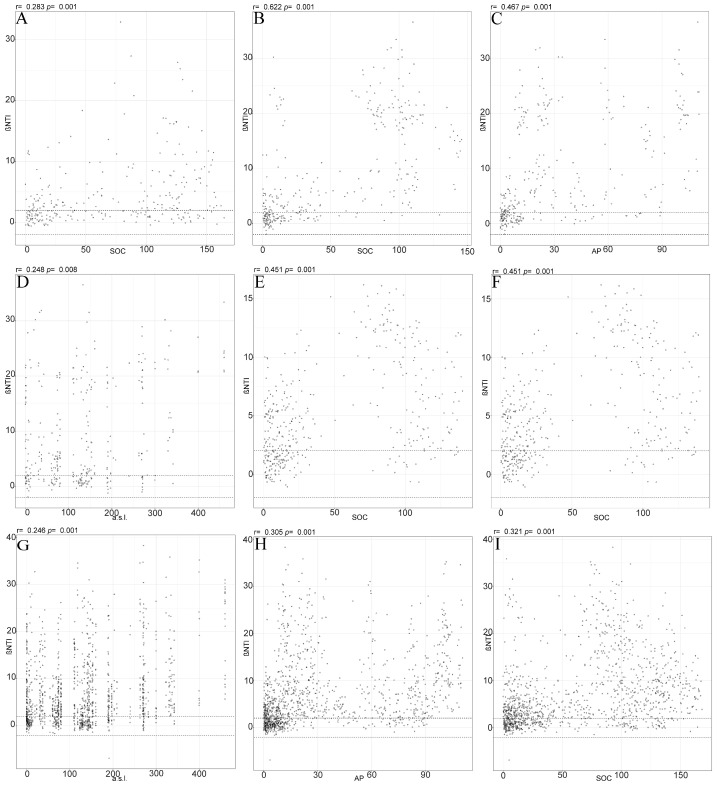
Correlation analysis between βNTI (β-nearest taxon index) and environmental factors for microbial community assembly in biological soil crusts (BSCs). (**A**–**D**) Correlations in surface and subsoil samples: (**A**) SOC (soil organic carbon) is positively correlated with βNTI in surface samples; (**B**–**D**) SOC, AP (available phosphorus), and a.s.l (elevation above sea level) is positively correlated with βNTI in subsoil samples. (**E**,**F**) Seasonal effects in May: SOC and AP positively influenced βNTI, enhancing environmental selection. (**G**–**I**) Seasonal effects in September: SOC, AP, and elevation are significantly correlated with βNTI, with nutrient limitations increasing selection pressures. Each scatterplot shows correlation coefficients (r) and significance levels (*p*).

A correlation analysis between the βNTI and environmental factors showed that microbial community assembly in the BSCs was significantly influenced by soil organic carbon (SOC), the organic matter concentration, and available phosphorus (AP). In the surface BSC samples, SOC was positively correlated with the βNTI (r = 0.283, *p* = 0.001), indicating its role in driving community variation through environmental selection (Figure 5A). In subsoil samples, SOC (r = 0.622, *p* = 0.001) and AP (r = 0.467, *p* = 0.001) showed significant positive correlations with the βNTI (Figure 5B–D). Elevation (r = 0.248, *p* = 0.008) also significantly affected the βNTI, thus reflecting the influence of geographic location on community assembly. SOC and AP were positively correlated with the βNTI in May (Figure 5E,F), suggesting that higher nutrient levels during the dry season enhanced community variation selection. In September, elevation, AP, and SOC were significantly positively correlated with the βNTI (Figure 5G–I), as nutrient limitations and geographic factors increased selection pressure during the wet season. The organic matter concentration also influenced community assembly in September, but pH and total nitrogen did not show a significant effect at any of the sites during any season (Figures S8–S11). Overall, SOC, AP, and elevation were key environmental factors that drove changes in the bacterial community βNTI, with seasonal variations also modulating community assembly processes.

## 4. Discussion

### 4.1. Bacterial Diversity and Composition of BSCs and Their Subsoils Across Different Sites

The microbial diversity and community composition of the BSCs were influenced by the crust type, soil layer, geographic location, and seasonal factors [28]. Subsoil samples of moss-BSCs and lichen-BSCs had significantly higher Shannon diversity indices than the surface BSC samples, suggesting that subsoil conditions, such as the high moisture and organic matter levels, created a more stable environment conducive to maintaining microbial diversity [29,30]. Moss-BSCs exhibited the highest species richness, as indicated by Chao1 and ACE indices, while cyano-BSCs had the lowest richness, likely due to the their nutrient-limited conditions [31]. Moss- and lichen-BSCs were richer in organic carbon and moisture than cyano-BSCs, and therefore provided more nutrients for microbial growth, which supported higher microbial diversity [32]. This observation supports our hypothesis that environmental factors such as moisture and nutrient availability play a key role in shaping microbial diversity, showing higher diversity in areas with favorable environmental conditions (e.g., Kubuqi and Tengger Deserts). These differences were likely linked to the more favorable moisture and nutrient conditions in the Kubqi and Tengger Deserts [33].

Seasonal factors also shaped microbial diversity and composition. The diversity indices were higher during the dry season (May), while the wet season (September) did not significantly increase the diversity. However, seasonal changes were evident in the community composition: Cyanobacteria were more abundant in samples collected during the dry season, while Clostridia and Proteobacteria increased significantly in samples collected during the wet season. This shift suggested that higher moisture availability during the wet season favored the growth of facultative anaerobes or anaerobes, such as Clostridia and Actinobacteria [34]. A PCoA showed that the surface and subsoil layers had distinct microbial community structures, with the surface BSC samples dominated by Cyanobacteria, particularly in cyano-BSCs, while the subsoil samples showed higher abundances of Clostridia and Actinobacteria, suggesting that deeper soils favored these groups [35]. Additionally, significant regional variation was observed. Samples from the Kubqi Desert had a higher Cyanobacterial abundance, while those from the Tengger Desert were enriched in Proteobacteria and Clostridia. These results highlight the importance of geographic location on shaping the microbial community composition, likely due to the differences in the moisture and nutrient availability [36,37]. Overall, the microbial diversity and composition of the BSCs were strongly influenced by the dynamic interplay among the BSC type, soil depth, geographic location, and seasonal factors, with each contributing to community structure and functional diversity.

### 4.2. Co-Occurrence Pattern Analysis of the Bacterial Communities

A network analysis revealed that the co-occurrence patterns of the microbial communities in the BSCs were shaped by several factors, including soil layer, BSC type, desert type, and seasonal variation. The surface BSC samples had more complex networks than the subsoil samples, with more nodes and edges indicating a strong, cooperative network structure, particularly the cyano-BSCs in which Cyanobacteria dominated. This suggests that surface microbial communities may rely on synergistic interactions and rapid adaptability to fluctuating environmental conditions, thus promoting resilience against short-term environmental stressors [38]. This pattern supports the hypothesis that microbial networks in the subsoil are more influenced by environmental selection, reflecting more stable conditions and localized interactions among microbial species, while surface layers, which are more dynamic, are shaped by stochastic processes. For example, the subsoil samples from moss- and lichen-BSCs had independent clusters of Proteobacteria, Actinobacteria, and Acidobacteria, indicating that microbial interactions in these environments were more constrained and specialized, likely as a response to resource-limited conditions.

The co-occurrence patterns also varied significantly across different BSC types. The cyano-BSC surface samples showed highly cooperative interactions, with Cyanobacteria forming tightly connected networks. These tightly interconnected networks in the surface BSCs suggest that Cyanobacteria may play a central role in nutrient cycling and microbial stability, efficiently sharing resources and responding to the harsh environmental conditions of the surface [38]. However, subsoil cyano-BSC samples indicated more complex and diverse interactions among the various bacterial phyla. Moss and lichen surface BSC samples showed dispersed networks with multiple independent modules, while the corresponding subsoil samples demonstrated greater modularity, particularly of Actinobacteria and Proteobacteria in moss BSCs and Actinobacteria and Acidobacteria in lichen-BSCs. These modular structures in the subsoil environments likely enhance microbial stability and allow for more efficient resource utilization, particularly under the nutrient-limited conditions typical of these environments. Seasonal and regional factors were found to further influence the microbial co-occurrence patterns. For example, in the Hunshandake Desert, moist conditions in May were associated with a higher network complexity, while the dry season in September was associated with more localized clustering of Acidobacteria and Actinobacteria. In the Kubqi and Tengger Deserts, samples from May showed tight networks dominated by Proteobacteria and Actinobacteria, while in samples from September, Chloroflexi and Clostridia were more prominent, reflecting a shift in microbial interactions under dry conditions. Overall, these findings suggested that the microbial communities in BSCs adjusted their interactions according to different seasonal and environmental conditions, and thus reflected their adaptation to fluctuations in moisture and nutrient availability.

### 4.3. Assembly Processes and Influencing Factors of Bacterial Communities in BSCs

The ecological assembly processes of bacterial communities in the BSCs were shaped by a combination of factors, including soil layer, BSC type, desert type, and environmental conditions, with significant differences in the contributions of stochastic processes and environmental selection, environmental selection and stochastic processes jointly drove bacterial community assembly. A βNTI analysis indicated that surface BSC samples were influenced by a balance of stochastic processes (dispersal limitation and drift) and environmental selection, with βNTI values generally in the range of −2 to 2, but stochastic processes dominated. This balance was likely driven by the dynamic and fluctuating surface environment, in which factors such as moisture and light intensity fluctuated, promoting both stochasticity and environmental filtering, due to the limited resources and greater external influences (such as precipitation and wind), and microbial colonization often depends on incidental factors, making random processes dominant in community assembly. During the dry season, the rapid changes in moisture further enhanced the role of random processes, while the rainy season may increase resource availability, thus strengthening the influence of environmental selection on surface communities [39]. This finding aligns with our hypothesis that environmental selection dominates in subsoil microbial communities, where stable resource conditions promote selective pressure, while surface soils exhibit more stochastic processes due to fluctuating conditions. Subsurface soils are relatively stable, with minimal temperature and moisture fluctuations, and prolonged resource competition. This stability make microbial communities more dependent on environmental selection, reducing the influence of random processes and promoting community assembly through resource availability and competitive exclusion. These findings were further supported by the RCbray results, which showed that stochastic processes were more prevalent in the surface BSC samples, while the subsoil samples were largely shaped by environmental selection, reflecting the subsoil stability and nutrient concentration [40].

Different types of BSCs demonstrated distinct assembly processes. In cyano-BSCs, stochastic processes dominated community assembly, whereas moss- and lichen-BSCs were more influenced by environmental selection, particularly in the subsoil samples. The dominance of stochastic processes in cyano-BSCs likely reflects the surface’s unstable conditions, where community assembly is more influenced by random dispersal and local environmental disturbances. In contrast, moss- and lichen-BSCs, with more stable and nutrient-rich subsoil environments, are shaped by environmental filtering that selects for specialized microbial groups adapted to these conditions [41]. The desert type also significantly affected assembly processes. For example, microbial communities in the Hunshandake Desert were more influenced by environmental selection because of the higher nutrient availability and more humid conditions of the desert. In contrast, communities in the Kubqi and Tengger Deserts were shaped more by stochastic processes, particularly under the arid conditions of the dry season. Seasonal changes also indirectly affected microbial assembly, in which resource availability was critical. Higher nutrient levels during the wet season (May) promoted environmental selection, while reduced resource availability during the dry season (September) promoted stochastic processes. Key environmental factors, such as SOC, AP, and elevation, also shaped community assembly [40]. High levels of subsoil organic carbon and phosphorus were associated with increased community heterogeneity, while the dynamic surface environments with lower nutrient levels were under a greater influence of stochastic processes. Overall, these findings highlight the complex yet flexible assembly processes of microbial communities in BSCs, with stochastic processes more prominent in the surface soils and environmental selection dominating in the subsoils, driven by resource availability and environmental stability.

## 5. Conclusions

This study highlighted the complex assembly of bacterial communities in BSCs shaped by both deterministic and stochastic processes. Environmental selection predominantly governed microbial community assembly in subsoil layers, with soil organic carbon, available phosphorus, and moisture availability driving community composition. In contrast, surface layers were under a greater influence of stochastic processes, with fluctuating moisture and light conditions promoting community variation. BSC type, soil depth, and geographic location were key determinants of microbial diversity, with moss- and lichen-BSCs exhibiting higher diversity than cyano-BSCs. Seasonal shifts also affected the diversity, with Cyanobacteria dominant in the dry season and Clostridia and Proteobacteria increasing during wetter periods. A co-occurrence network analysis revealed that surface BSC samples had greater complexity with cooperative interactions, while subsoil samples displayed more modular and specialized networks. Overall, this study provided valuable insights into the dynamic processes that shape microbial communities in desert ecosystems, emphasizing the role of environmental factors and seasonal changes that drive microbial diversity and interactions.

## Data Availability

The original contributions presented in this study are included in the article/Supplementary Materials. Further inquiries can be directed to the corresponding authors.

## Supplementary Materials

The following supporting information can be downloaded at: https://www.mdpi.com/article/10.3390/microorganisms13020446/s1, Figure S1: Alpha diversity indices of bacterial communities in different biological soil crust (BSC) types and their subsoils; Figure S2: Alpha diversity indices of bacterial communities in biological soil crusts (BSCs) across different regions and seasons; Figure S3: Principal Coordinates Analysis (PCoA) of bacterial community composition in different biological soil crust (BSC) types and subsoils across various regions and seasons; Figure S4: Principal Coordinates Analysis (PCoA) of bacterial community composition across different regions and seasons in biological soil crusts (BSCs); Figure S5: Phylum-level composition of bacterial communities in different BSC types and their subsoils; Figure S6: Phylum-level composition of bacterial communities in BSC samples from different deserts and sampling times; Figure S7: Co-occurrence network analysis of bacterial communities in different types of BSCs; Figure S8: Correlation analysis between βNTI of bacterial communities and environmental factors in surface BSC samples; Figure S9: Correlation analysis between βNTI of bacterial communities and environmental factors in subsoil samples; Figure S10: Correlation analysis between βNTI of bacterial communities and environmental factors in may BSC samples; Figure S11: Correlation analysis between βNTI of bacterial communities and environmental factors in september BSC samples; Table S1: Collection Months, Geographic Locations, and Physicochemical Properties of 50 BSC Samples; Table S2: The network parameters for samples from different crust samples.
