# Supplementary material for: Spatiotemporal Dynamics of Bacterial Community Assembly and Co-Occurrence Patterns in Biological Soil Crusts of Desert Ecosystems
Source: Microorganisms. 2025 Feb 18;13(2):446. doi: 10.3390/microorganisms13020446 (PMC11858719; doi:10.3390/microorganisms13020446)
Supplement: Supplementary file 1 [file microorganisms-13-00446-s001.zip › Supplementary materials.pdf]

Supporting information

Figure S1. Alpha diversity indices of bacterial communities in different biological soil crust (BSC) types and their subsoils.

The violin plots display the ACE, Chao1, Inverse Simpson, Pielou evenness, Richness, Simpson evenness, and Shannon indices for Cyano-BSCs (C), subsoils of Cyano-BSCs (Cs), Lichen-BSCs (L), subsoils of Lichen-BSCs (Ls), Moss-BSCs (M), and subsoils of Moss-BSCs (Ms). Different letters above the plots indicate significant differences among groups ($p < 0.05$).

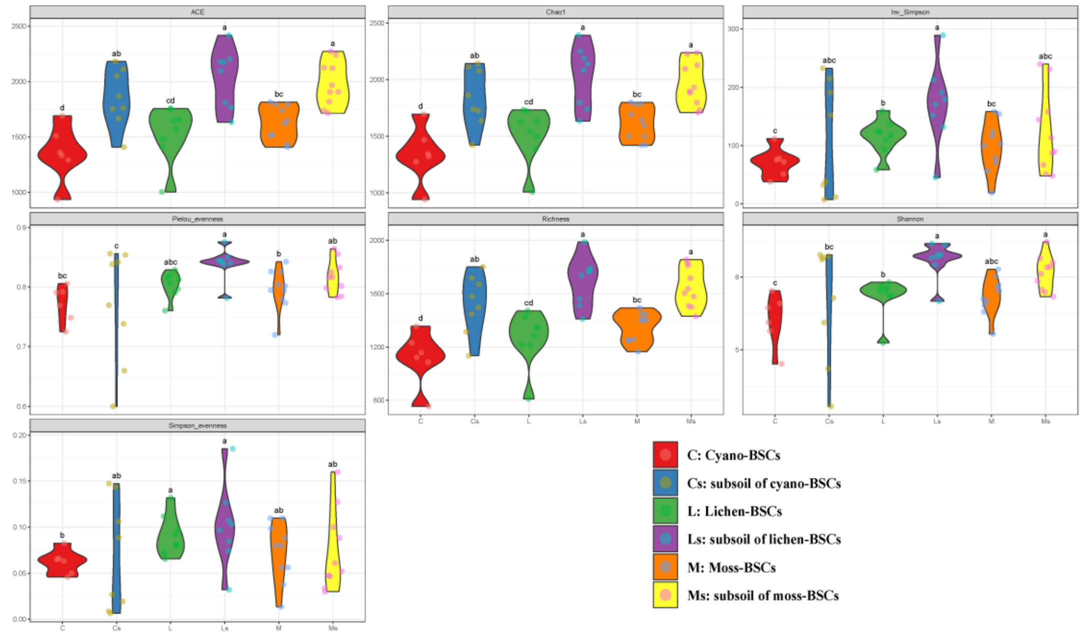

Figure S2. Alpha diversity indices of bacterial communities in biological soil crusts (BSCs) across different regions and seasons.

The violin plots display ACE, Chao1, Inverse Simpson, Pielou evenness, Richness, Simpson evenness, and Shannon indices for samples collected in May and September from Hunshandake Sandy Land (mH, sH), Kubuqi Desert (mK, sK), and Tengger Desert (mT, sT). Different letters above the plots indicate significant differences among groups ($p < 0.05$).

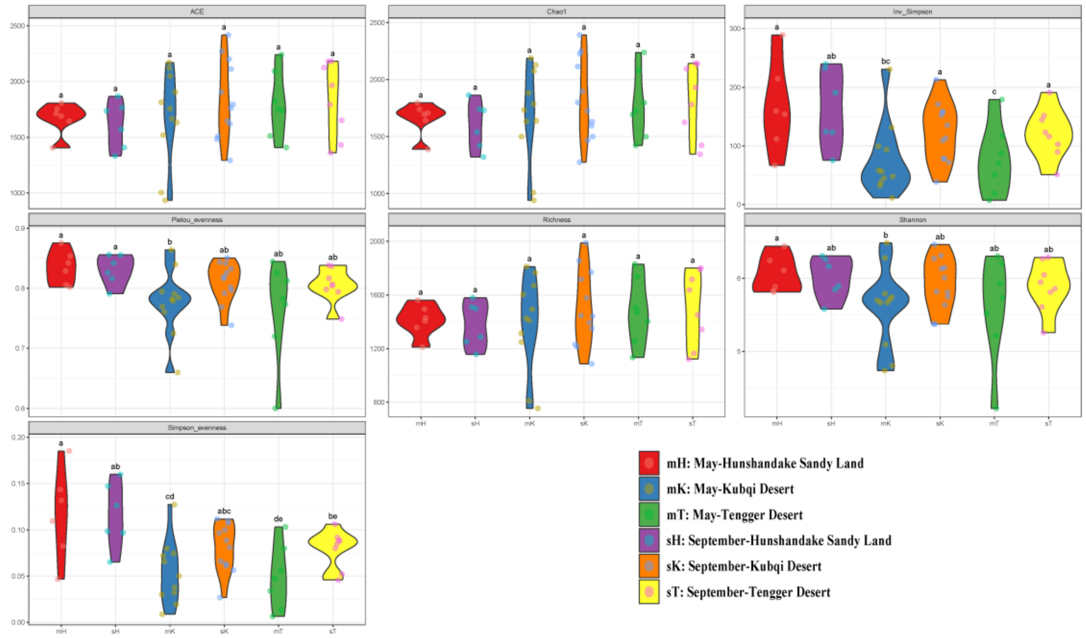

Figure S3. Principal Coordinates Analysis (PCoA) of bacterial community composition in different biological soil crust (BSC) types and subsoils across various regions and seasons. The analysis is based on Bray-Curtis dissimilarity, with PCoA1 and PCoA2 explaining 25.81% and 17.37% of the variation, respectively. Different groups include Cyano-BSCs (C), subsoils of Cyano-BSCs (Cs), Lichen-BSCs (L), subsoils of Lichen-BSCs (Ls), Moss-BSCs (M), and subsoils of Moss-BSCs (Ms). Samples are color-coded by group, and ellipses represent 95% confidence intervals for each group. ANOSIM results (R = 0.462, p = 0.001) indicate significant differences in bacterial community composition among groups, highlighting the influence of crust type, soil depth, region, and season on microbial communities.

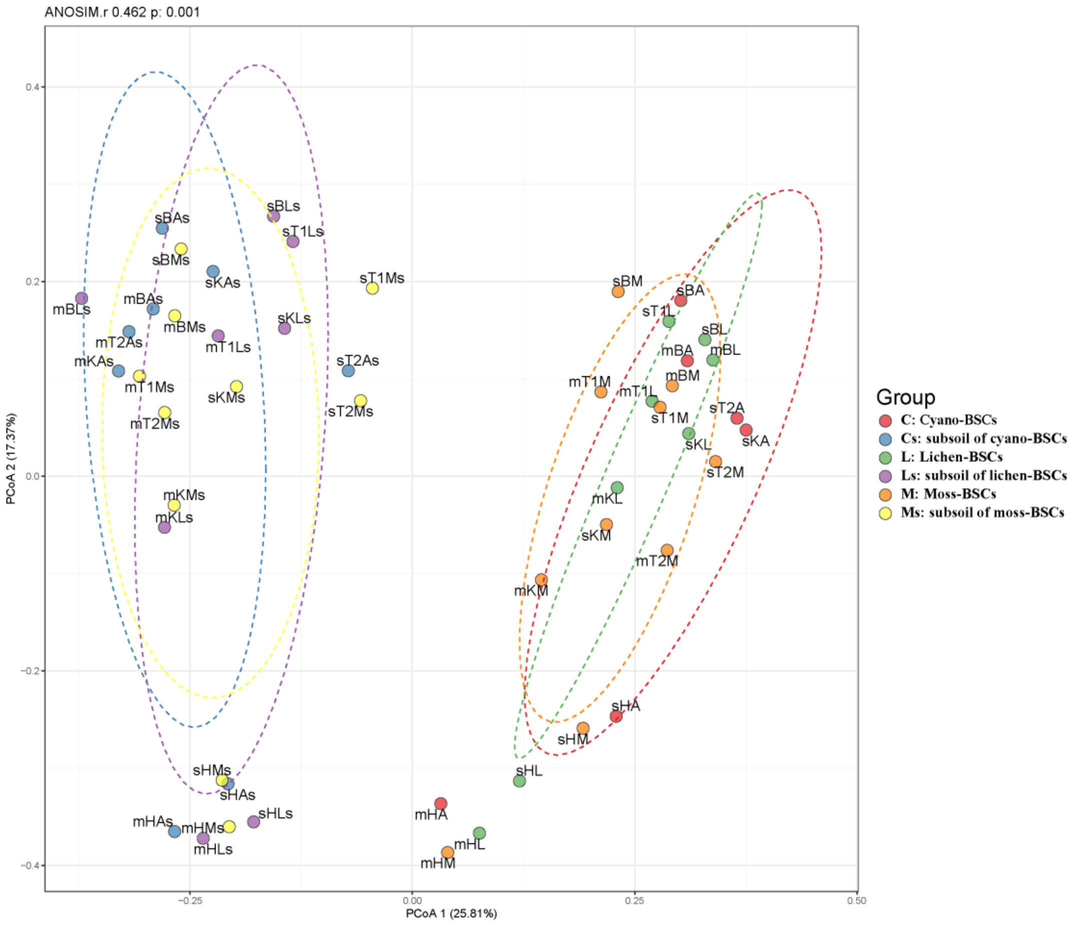

Figure S4. Principal Coordinates Analysis (PCoA) of bacterial community composition across different regions and seasons in biological soil crusts (BSCs).

The analysis is based on Bray-Curtis dissimilarity, with PCoA1 and PCoA2 explaining 25.81% and 17.37% of the variation, respectively. Groups include samples collected in May (mH, mK, mT) and September (sH, sK, sT) from Hunshandake Sandy Land (H), Kubuqi Desert (K), and Tengger Desert (T). Samples are color-coded by group, and ellipses represent 95% confidence intervals for each group.

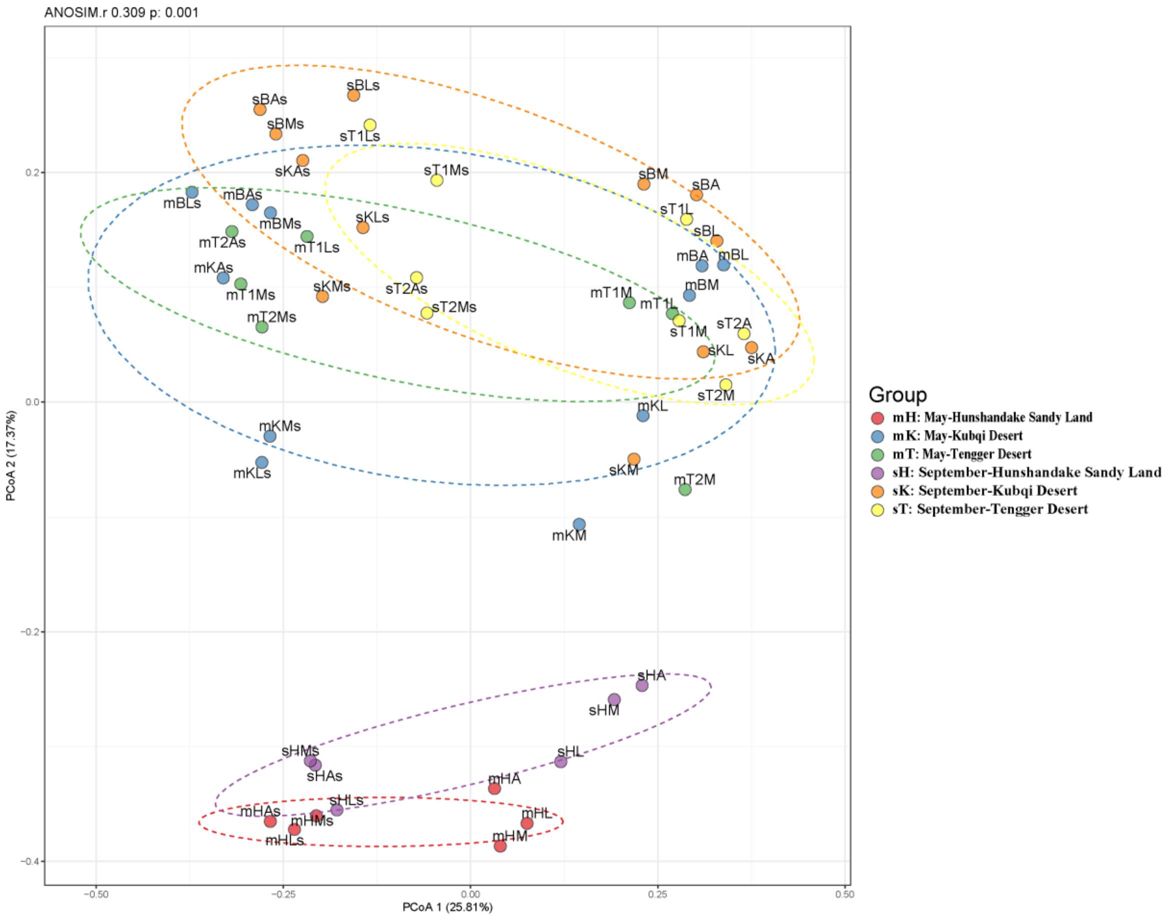

Figure S5. Phylum-level composition of bacterial communities in different BSC types and their subsoils.

A: Cyanobacterial crust; L: Lichen crust; M: Moss crust; s: Subsoil samples

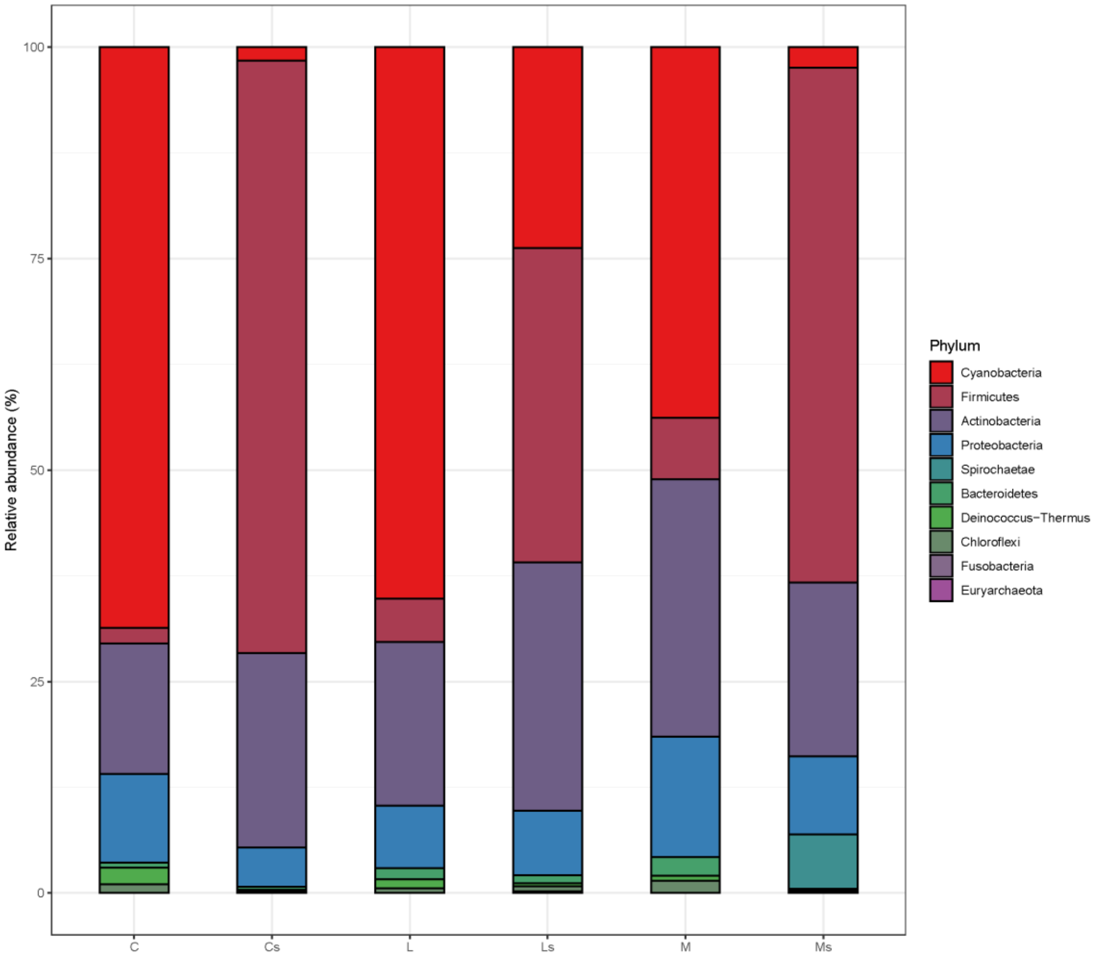

Figure S6. Phylum-level composition of bacterial communities in BSC samples from different deserts and sampling times.

The first letter of the sample identifier represents the sampling time: m for May, s for September; H for Hunshandake Sandy Land, K for Kubqi Desert, T for Tengger Desert.

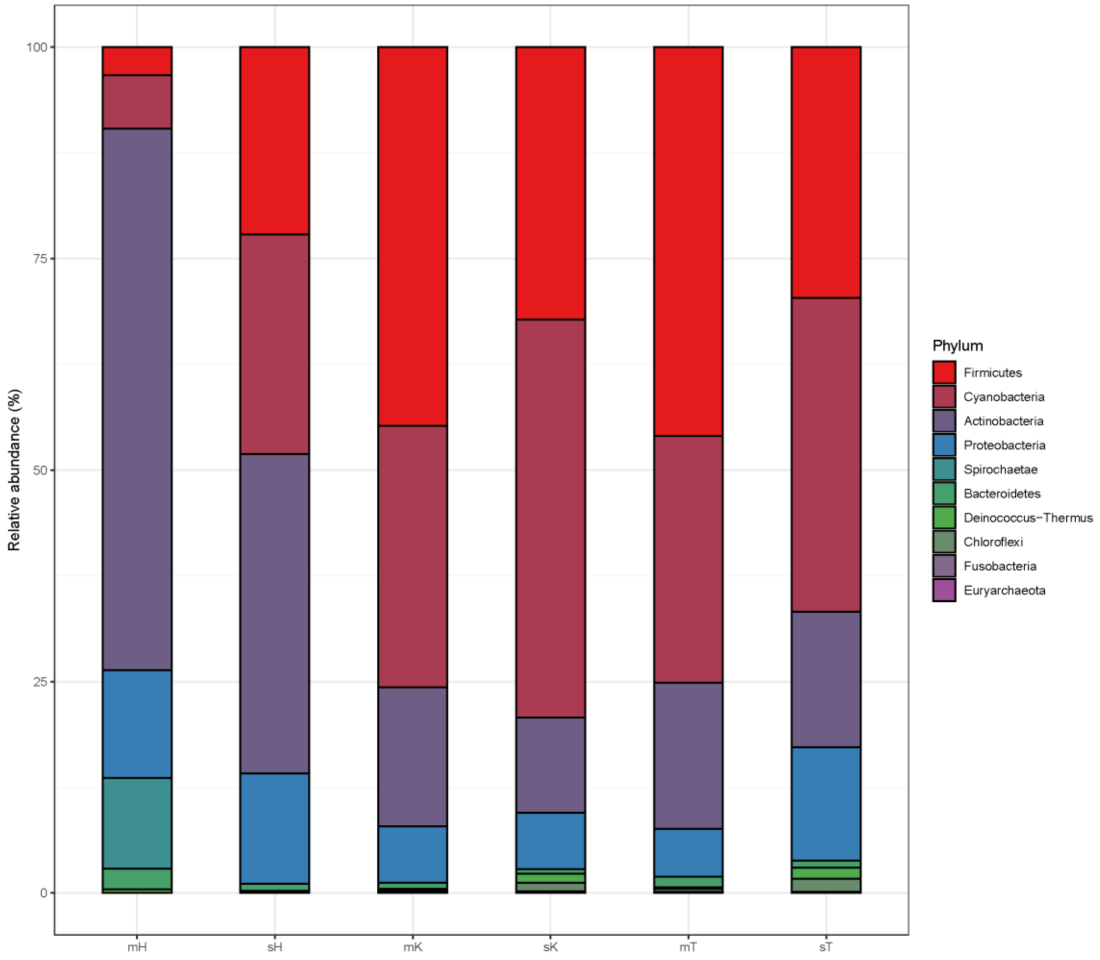

Figure S7. Co-occurrence network analysis of bacterial communities in different types of BSCs. The networks represent bacterial communities in May and September from Hunshandake Sandy Land (mH, sH), Kubuqi Desert (mK, sK), and Tengger Desert (mT, sT). Nodes represent bacterial taxa at the phylum level, with colors indicating different phyla, and edges represent significant correlations between taxa (Spearman's correlation, $p < 0.001$, $|r| > 0.80$). Edge thickness corresponds to correlation strength. The networks illustrate regional and seasonal variations in microbial community structure, with differences in network complexity and modularity between sampling locations and periods.

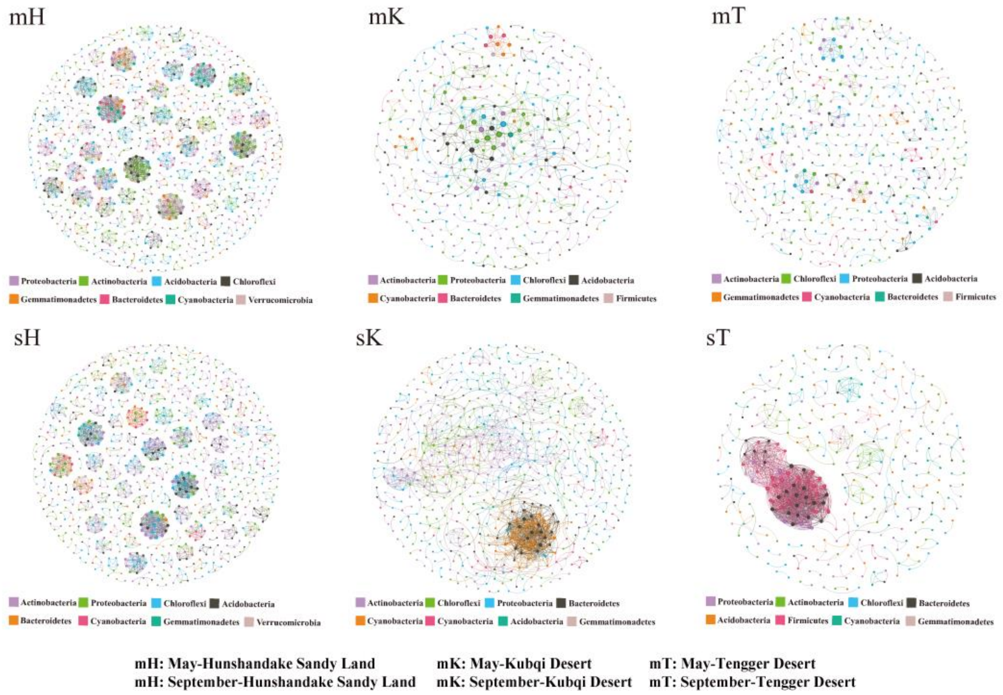

Figure S8. Correlation analysis between βNTI of bacterial communities and environmental factors in surface BSC samples.

The plots represent the relationships between βNTI and altitude (a.s.l.), pH, nitrate nitrogen (AN), available phosphorus (AP), chlorophyll α (Chlα), and soil organic carbon (SOC), with R values and p values displayed at the top of each plot.

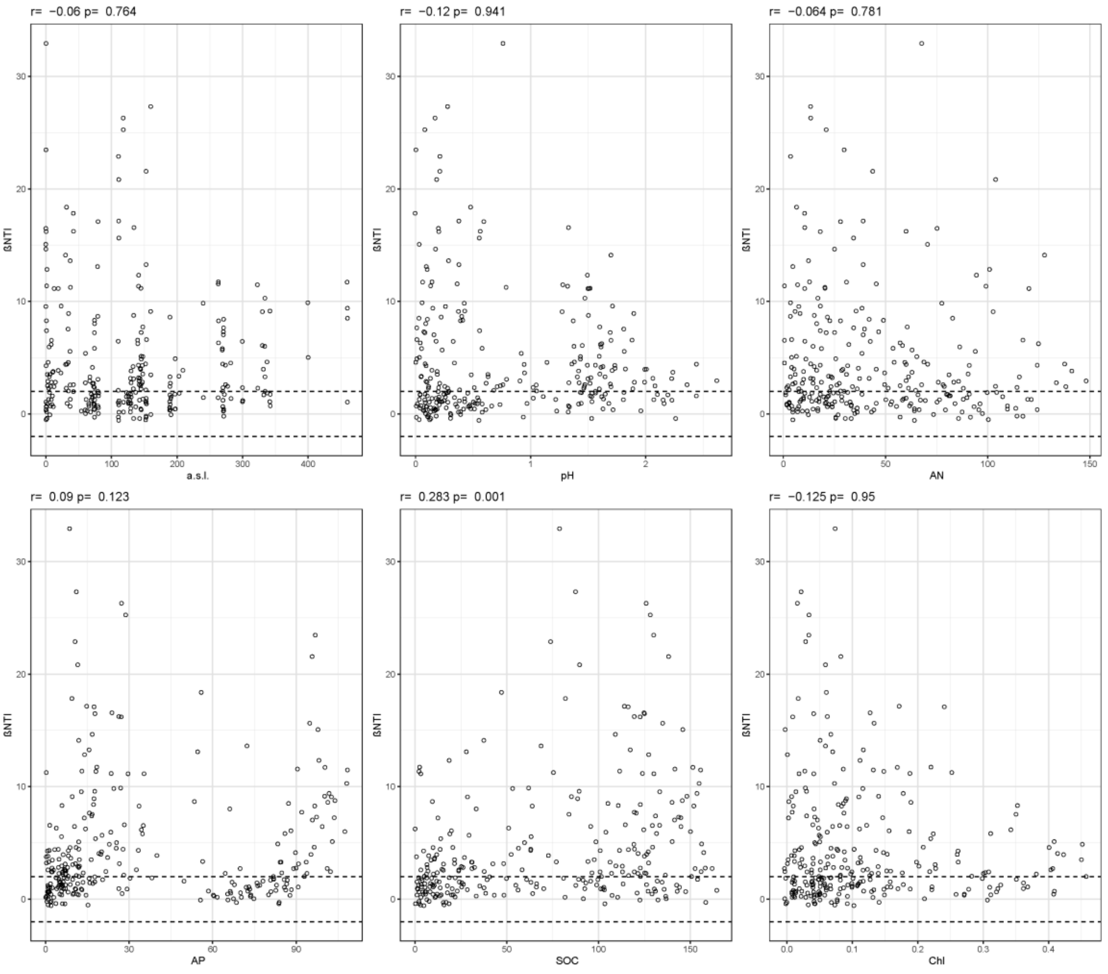

Figure S9. Correlation analysis between βNTI of bacterial communities and environmental factors in subsoil samples.

The plots represent the relationships between βNTI and altitude (a.s.l.), pH, nitrate nitrogen (AN), available phosphorus (AP), chlorophyll α (Chlα), and soil organic carbon (SOC), with R values and p values displayed at the top of each plot.

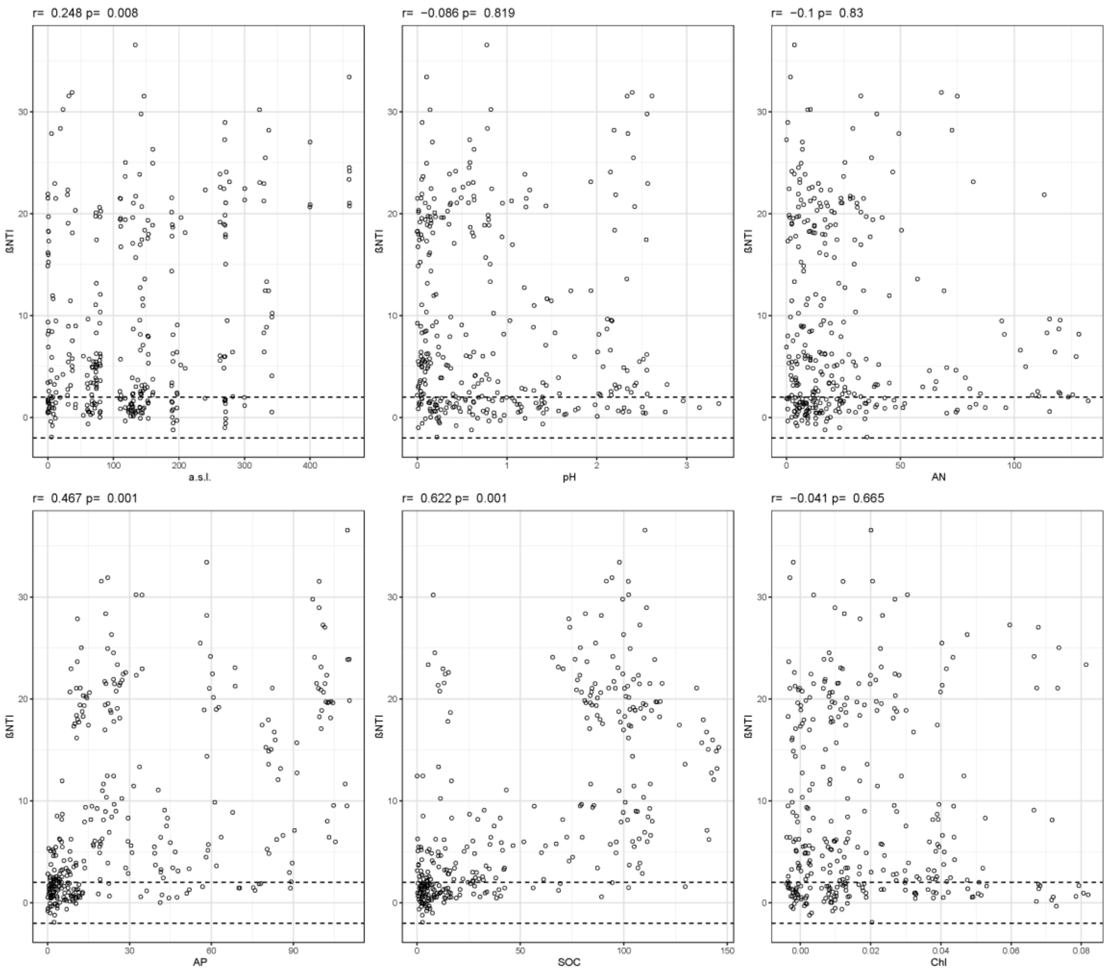

Figure S10. Correlation analysis between βNTI of bacterial communities and environmental factors in may BSC samples.

The plots represent the relationships between βNTI and altitude (a.s.l.), pH, nitrate nitrogen (AN), available phosphorus (AP), chlorophyll α (Chlα), and soil organic carbon (SOC), with R values and p values displayed at the top of each plot.

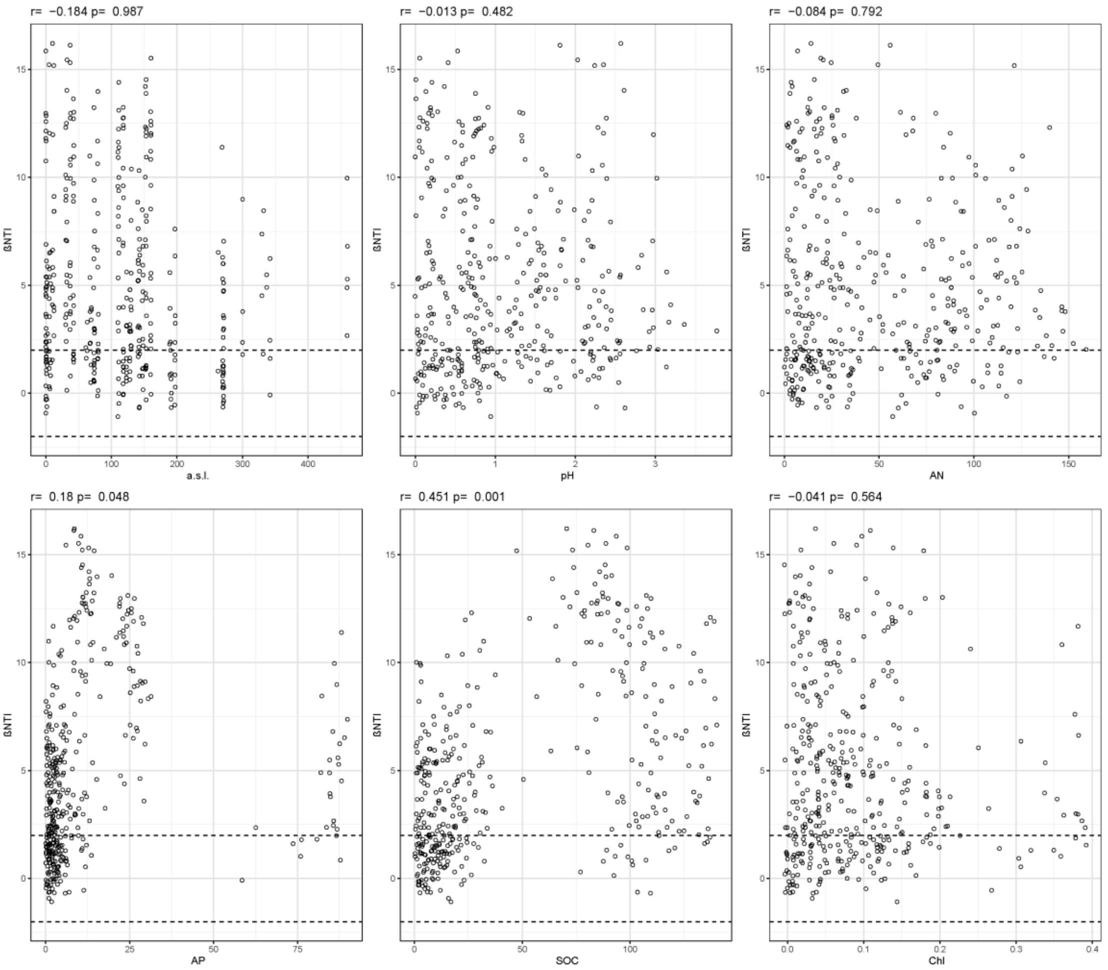

Figure S11. Correlation analysis between βNTI of bacterial communities and environmental factors in september BSC samples.

The plots represent the relationships between βNTI and altitude (a.s.l.), pH, nitrate nitrogen (AN), available phosphorus (AP), chlorophyll α (Chlα), and soil organic carbon (SOC), with R values and p values displayed at the top of each plot.

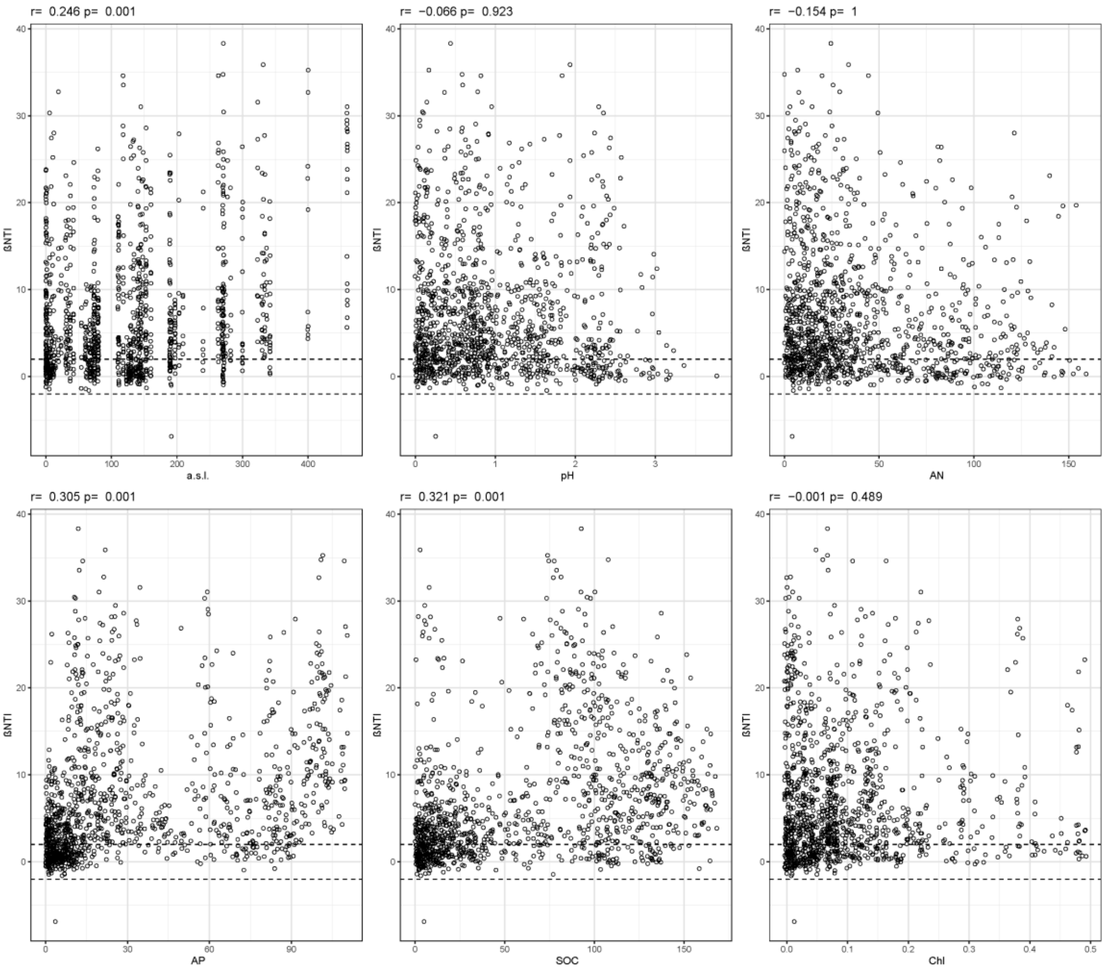

Table S1. Collection Months, Geographic Locations, and Physicochemical Properties of 50 BSC Samples

The sample labels indicate the collection month and location. The first letter refers to the sampling month: m for May and s for September. Samples labeled with "A" were collected from the eastern region (Hunshandake Desert), "B" from the central region (Kubuqi Desert), and "C" from the western region (Tengger Desert). Geographic information includes longitude (lng), latitude (lat), and altitude (asl). Meteorological data include mean annual precipitation (MAP), mean annual temperature (MAT), humidity (HU), and annual sunshine duration (ASD). The physicochemical properties include available phosphorus (AP), available nitrogen (AN), soil organic carbon (SOC), and chlorophyll α (Chl α) content. The values for the physicochemical properties are presented as the mean ± standard deviation from three independent experiments.

**Sites**	**Samples**	**Geographic location**			**Meteorological data**				**Physicochemical properties**				
		Lng	Lat	a.s.l. (m)	MAP (mm)	MAT (ºC)	HU (%)	ASD (h/year)	pH	AN ($mg\cdot kg^{-1}$)	AP ($mg\cdot kg^{-1}$)	SOC ($g\cdot kg^{-1}$)	Chl *a* ($g\cdot g^{-1}$)
A	mHA	42.4265	116.769	1387	351	1.83	58.21	2887	6.89 ± 0.03	138.83 ± 3.64	16.17 ± 0.23	36.67 ± 0.46	0.09 ± 0.02
	mHAs	42.4265	116.769	1387	351	1.83	58.21	2887	6.89 ± 0.02	100.67 ± 1.01	13.52 ± 0.09	16.74 ± 0.17	0.03 ± 0.01
	mHL	42.4266	116.769	1382	351	1.83	58.21	2887	6.26 ± 0.09	115.5 ± 1.75	17.33 ± 0.58	113.7 ± 0.30	0.04 ± 0.02
	mHLs	42.4266	116.769	1382	351	1.83	58.21	2887	6.67 ± 0.05	65.33 ± 1.75	15.88 ± 0.00	19.49 ± 0.43	0.05 ± 0.01
	mHM	42.4272	116.766	1380	351	1.83	58.21	2887	7.00 ± 0.07	172.67 ± 1.75	10.00 ± 0.53	137.49 ± 0.20	0.19 ± 0.02
	mHMs	42.4272	116.766	1380	351	1.83	58.21	2887	7.07 ± 0.02	145.83 ± 1.75	8.23 ± 0.27	33.41 ± 0.10	0.05 ± 0.01
	sHA	42.4257	116.769	1373	351	1.83	58.21	2887	6.99 ± 0.02	62.99 ± 2.67	4.24 ± 0.47	10.51 ± 0.89	0.17 ± 0.02
	sHAs	42.4257	116.769	1373	351	1.83	58.21	2887	8.46 ± 0.00	22.16 ± 2.67	3.16 ± 0.49	8.70 ± 0.26	0.00 ± 0.00
	sHL	42.4265	116.769	1384	351	1.83	58.21	2887	6.89 ± 0.02	84.57 ± 3.64	5.18 ± 0.49	16.04 ± 0.46	0.49 ± 0.02
	sHLs	42.4265	116.769	1384	351	1.83	58.21	2887	7.79 ± 0.02	34.99 ± 1.01	4.01 ± 0.23	6.15 ± 0.46	0.01 ± 0.00
	sHM	42.4273	116.766	1384	351	1.83	58.21	2887	6.80 ± 0.18	53.07 ± 2.02	4.50 ± 0.40	11.34 ± 0.46	0.23 ± 0.02
	sHMs	42.4273	116.766	1380	351	1.83	58.21	2887	8.03 ± 0.05	17.49 ± 2.02	3.08 ± 0.31	4.00 ± 0.10	0.01 ± 0.00
B	mKAs	39.3728	109.501	1510	245	6.88	46.3	2933	10.03 ± 0.03	13.41 ± 2.67	12.59 ± 0.40	5.55 ± 0.34	0.00 ± 0.00
	mKL	39.3728	109.501	1510	245	6.88	46.3	2933	8.42 ± 0.04	31.49 ± 0.71	10.85 ± 1.55	12.49 ± 0.47	0.12 ± 0.02

	mKLs	39.3728	109.501	1510	245	6.88	46.3	2933	9.18 ± 0.02	26.24 ± 2.41	13.56 ± 0.55	7.96 ± 0.08	0.01 ± 0.01
	mKM	39.3728	109.501	1510	245	6.88	46.3	2933	8.33 ± 0.07	39.07 ± 3.12	12.33 ± 1.46	14.72 ± 0.15	0.13 ± 0.02
	mKMs	39.3728	109.501	1510	245	6.88	46.3	2933	8.65 ± 0.02	25.66 ± 0.34	12.13 ± 0.74	11.17 ± 0.11	0.08 ± 0.02
	sKA	40.308	108.404	1050	338	6.88	46.3	2933	8.45 ± 0.02	114.33 ± 4.77	98.11 ± 1.39	117.62 ± 0.47	0.03 ± 0.01
	sKAs	40.308	108.404	1050	338	6.88	46.3	2933	8.60 ± 0.02	31.5 ± 4.37	37.69 ± 0.44	16.62 ± 1.47	0.00 ± 0.00
	sKL	40.25	108.365	1110	245	6.88	46.3	2933	8.36 ± 0.04	35.00 ± 0.18	37.83 ± 1.37	74.40 ± 1.60	0.09 ± 0.01
	sKLs	40.25	108.365	1110	245	6.88	46.3	2933	8.82 ± 0.02	18.67 ± 0.05	113.67 ± 0.36	85.07 ± 2.02	0.01 ± 0.00
	sKM	40.435	108.403	1050	245	6.88	46.3	2933	8.27 ± 0.02	70.00 ± 1.39	112.65 ± 2.00	166.12 ± 11.57	0.30 ± 0.02
	sKMs	40.435	108.403	1050	245	6.88	46.3	2933	9.08 ± 0.04	28.00 ± 2.77	71.80 ± 0.47	105.82 ± 1.96	0.01 ± 0.00
	mBA	40.2256	107.148	1240	155	10.1	45.58	3008	8.88 ± 0.05	55.41 ± 5.14	11.52 ± 0.84	4.23 ± 9.24	0.07 ± 0.01
	mBAs	40.2256	107.148	1240	155	10.1	45.58	3008	9.19 ± 0.03	49.57 ± 1.12	14.47 ± 0.37	0.45 ± 2.09	0.01 ± 0.00
	mBL	40.2263	107.147	1239	155	10.1	45.58	3008	8.52 ± 0.02	148.74 ± 4.15	10.34 ± 0.72	10.25 ± 0.09	0.08 ± 0.01
	mBLs	40.2263	107.147	1239	155	10.1	45.58	3008	9.23 ± 0.04	35.57 ± 2.03	13.45 ± 0.50	1.65 ± 0.11	0.01 ± 0.00
	mBM	40.225	107.148	1239	155	10.1	45.58	3008	8.49 ± 0.19	48.41 ± 1.94	11.31 ± 0.90	26.08 ± 0.58	0.11 ± 0.01
	mBMs	40.225	107.148	1239	155	10.1	45.58	3008	9.09 ± 0.01	60.66 ± 1.41	11.06 ± 0.56	3.43 ± 0.00	0.01 ± 0.00
	sBA	40.2256	107.148	1240	155	10.1	45.58	3008	8.62 ± 0.02	47.83 ± 2.03	24.37 ± 1.56	137.58 ± 0.27	0.08 ± 0.01
	sBAs	40.2256	107.148	1240	155	10.1	45.58	3008	9.23 ± 0.01	25.67 ± 1.32	113.01 ± 0.71	118.88 ± 0.23	0.02 ± 0.00
	sBL	40.2263	107.147	1239	155	10.1	45.58	3008	8.32 ± 0.07	73.50 ± 1.85	28.09 ± 2.58	135.28 ± 0.90	0.04 ± 0.01
	sBLs	40.2263	107.147	1239	155	10.1	45.58	3008	9.09 ± 0.01	50.17 ± 1.06	24.06 ± 0.95	103.85 ± 0.56	0.01 ± 0.00
	sBM	40.225	107.148	1239	155	10.1	45.58	3008	8.49 ± 0.07	78.17 ± 1.41	108.16 ± 1.36	156.15 ± 0.23	0.08 ± 0.01
	sBMs	40.225	107.148	1239	155	10.1	45.58	3008	9.22 ± 0.07	43.17 ± 1.23	94.44 ± 1.00	146.29 ± 0.23	0.01 ± 0.00
	mT1L	38.6753	105.624	1350	110	8.32	44.86	3083	7.94 ± 0.09	112.57 ± 1.41	13.30 ± 0.22	21.2 ± 0.56	0.21 ± 0.01
	mT1Ls	38.6753	105.624	1350	110	8.32	44.86	3083	9.23 ± 0.02	37.91 ± 1.15	11.41 ± 0.27	6.35 ± 0.06	0.04 ± 0.00
C	mT1M	38.6757	105.625	1392	110	8.32	44.86	3083	8.70 ± 0.01	34.41 ± 2.21	12.43 ± 0.21	17.99 ± 0.18	0.16 ± 0.01
	mT1Ms	38.6757	105.625	1392	110	8.32	44.86	3083	9.45 ± 0.09	26.24 ± 1.59	10.55 ± 0.13	5.21 ± 0.05	0.01 ± 0.00
	sT1L	38.6753	105.624	1350	110	8.32	44.86	3083	8.70 ± 0.02	44.85 ± 1.15	21.92 ± 0.30	99.96 ± 1.00	0.14 ± 0.01

sT1Ls	38.6753	105.624	1350	110	8.32	44.86	3083	9.28 ± 0.04	32.67 ± 1.32	35.54 ± 0.10	110.99 ± 1.11	0.03 ± 0.00
sT1M	38.6757	105.625	1392	110	8.32	44.86	3083	8.50 ± 0.02	52.5 ± 1.59	39.62 ± 0.46	140.67 ± 1.41	0.15 ± 0.02
sT1Ms	38.6757	105.625	1392	110	8.32	44.86	3083	9.24 ± 0.02	51.33 ± 1.32	24.42 ± 0.37	90.11 ± 0.90	0.01 ± 0.00
mT2As	38.6228	105.559	1319	110	8.32	44.86	3083	9.43 ± 0.01	30.32 ± 2.12	10.03 ± 0.03	2.91 ± 0.90	0.00 ± 0.00
mT2M	38.6245	105.559	1313	110	8.32	44.86	3083	7.91 ± 0.02	24.49 ± 1.68	22.19 ± 0.31	24.53 ± 0.25	0.39 ± 0.04
mT2Ms	38.6245	105.559	1313	110	8.32	44.86	3083	9.11 ± 0.19	20.19 ± 1.15	9.13 ± 1.01	1.31 ± 0.01	0.00 ± 0.00
sT2A	38.6228	105.559	1319	110	8.32	44.86	3083	8.22 ± 0.19	51.33 ± 1.56	77.82 ± 0.87	147.09 ± 1.41	0.08 ± 0.01
sT2As	38.6228	105.559	1319	110	8.32	44.86	3083	9.19 ± 0.02	30.33 ± 0.71	32.58 ± 0.47	112.93 ± 1.68	0.01 ± 0.00
sT2M	38.6245	105.559	1313	110	8.32	44.86	3083	8.41 ± 0.01	57.17 ± 1.36	94.24 ± 0.23	168.66 ± 1.15	0.08 ± 0.01
sT2Ms	38.6245	105.559	1313	110	8.32	44.86	3083	9.14 ± 0.19	40.83 ± 1.00	54.97 ± 0.25	43.68 ± 0.44	0.01 ± 0.00

Table S2. The network parameters for samples from different crust samples

including the number of nodes, number of edges, number of positive correlations, number of negative correlations, average degree, average path length, network diameter, network density, and clustering coefficient.

	nodes	edges	positive.cor	negative.cor	average degree	average path length	network diameter	network density	clustering coefficient
A	600	4452	4303	149	14.84	1	1	0.024775	1
L	422	3039	2986	53	14.40284	1.462076	5.940154	0.034211	0.980989
M	231	922	910	12	7.982684	1.49645	7.868947	0.034707	0.966209
As	445	4304	4281	23	19.34382	1.144148	3.968627	0.043567	0.996438
Ls	466	2774	2751	23	11.90558	1.643959	5.959322	0.025603	0.965436
Ms	346	2409	2394	15	13.92486	1.131733	6.857495	0.040362	0.978543
mH	888	2711	2343	368	6.105856	1	1	0.006884	1
mK	448	575	510	65	2.566964	6.442115	20.38505	0.005743	0.420354
mT	491	571	534	37	2.325866	1	1	0.004747	1
sH	357	1292	1274	18	7.238095	1.686531	7.945762	0.020332	0.894821
sK	645	1593	1539	54	4.939535	8.883452	27.81649	0.00767	0.626039
sT	357	1292	1274	18	7.238095	1.686531	7.945762	0.020332	0.894821
